# Novel reversible selective inhibitor of CRM1 for targeted therapy in ovarian cancer

**DOI:** 10.1186/s13048-015-0166-y

**Published:** 2015-06-10

**Authors:** Xuejiao Liu, Yulong Chong, Huize Liu, Yan Han, Mingshan Niu

**Affiliations:** Blood Diseases Institute, Xuzhou Medical College, Xuzhou, Jiangsu China; Insititute of Nervous System Diseases, Xuzhou Medical College, Xuzhou, Jiangsu China; Department of Hematology, Affiliated Hospital of Xuzhou Medical College, Xuzhou, Jiangsu China; Dalian Center for Disease Control and Prevention, Dalian, Liaoning China

**Keywords:** CRM1 inhibitor, S109, Ovarian cancer, Antitumor

## Abstract

**Background:**

Ovarian cancer represents the most fatal type of gynecological malignancies. Unfortunately, there are still no effective targeted treatment strategies for ovarian cancer. Overexpression of CRM1 has been correlated with poor prognosis of patients with ovarian cancer.

**Aim:**

In this study, we investigated the antitumor effects of a novel reversible inhibitor of CRM1 in ovarian cancer cells.

**Methods:**

The effects of S109 on proliferation was detected by CCK-8, EdU, clonogenic assay. The protein expression were determined by Western blot. The subcellular localization of RanBP1 was analyzed by immunofluorescence microscopy assay.

**Results:**

We demonstrated that S109 could induce nuclear accumulation of RanBP1, a canonical biomarker for CRM1 inhibition. This effect was clearly reversible in the majority of the cells, whereas the inhibitory effect of LMB could not be reversed. Our data reveal that treatment with S109 results in decrease in proliferation and colonogenic capacity of ovarian cancer cells by arresting cell cycle. Mechanistically, S109 treatment increase the expression of the cyclin-dependent kinase inhibitor p21, while it reduced the expression of cell cycle promoting proteins, Cyclin D1 and Cyclin B. CRM1 level itself was also down-regulated following S109 treatment. Furthermore, the nuclei of cells incubated with S109 accumulated tumor suppressor proteins (Foxo1, p27 and IκB-α). More importantly, Cys528 mutation of CRM1 abolished the ability of S109 to block proliferation of ovarian cancer cells.

**Conclusions:**

Together, our study identifies CRM1 as a valid target in ovarian cancer and provides a basis for the development of S109 in ovarian cancer.

## Introduction

Ovarian cancer is the most lethal gynecologic cancer and the fifth most common cause of cancer death in women [1]. Most of patients are usually detected at advanced stages, resulting in poor prognoses [2]. Despite many recent advancements in the field of ovarian cancer therapeutics, there has been minimal improvement in survival over the past 30 years. Surgical tumor debulking followed by chemotherapy with a combination of platinum-taxane as first line treatment for ovarian cancer [3]. However, most of patients will recur with chemotherapeutic-resistant tumors. Thus, new molecular targeted therapies are urgently needed.

The pathogenesis of ovarian cancer represents a multistep process that involves accumulation of multiple genetic and molecular lesions leading to the selection of a malignant clone [4]. The activation of the phosphatidylinositol 3 kinase (PI3K) pathway is frequently deregulated in up to 70 % of ovarian cancers through mechanisms that include amplification of *PIK3CA* and *AKT* or inactivating mutations of *PTEN* [5, 6]. The oncogenic activation of MAPK and NF-κB pathway is also associated with the pathogenesis of ovarian cancer [7, 8]. Unfortunately, despite a sound biological rationale and encouraging activity in preclinical models, the inhibitors of PI3K pathway have little effect in clinical trials [9]. Given the complexity and redundancy of the signaling network, development of new therapeutic approaches was needed, such as targeting multiple pathway simultaneously or combination with other targeted therapies.

Chromosomal region maintenance 1 (CRM1) is one of such attractive targets for anticancer therapy [10]. More recently, it has been reported that overexpression of CRM1 is correlated with poor prognosis in ovarian cancer [11]. Knockdown of CRM1 expression arrests cell cycle progression and inhibits the proliferation of ovarian cancer cells both in vitro and in vivo [12]. CRM1 is a key member of nuclear transport receptors and recognizes its export cargos through specific leucine-rich nuclear export signal (NES) consensus sequences [13]. CRM1 cargos include most of tumor suppressor proteins including Foxos, p53, p21, p27, APC, survivin and inhibitor of κB-α (IκB-α) [14]. Therefore, inhibiting CRM1 can target multiple pathway simultaneously and is a promising therapeutic target for ovarian cancer treatment.

An increasing number of compounds have been isolated or synthesized that inhibit CRM1 [15, 16]. However, most of them are irreversible inhibitors which have toxicity on normal cells. Leptomycin B (LMB) is the classic CRM1 inhibitor but is not found to be clinically useful due to severe toxicities [17]. This however did not deter the search for novel compounds, with reduced toxicities that could target nuclear export. More recently, it has been reported that SINE compounds are novel semi-reversible inhibitors of CRM1 to be developed for clinical use. SINE inhibitor (KPT-330) is generally well tolerated and can be administered over prolonged periods in several phase I clinical trials [18]. Therefore, the reversible inhibitor of CRM1 should be safe and well-tolerated in patients.

In this study, we investigated the effect of a novel reversible CRM1 inhibitor S109 on ovarian cancer. We found that S109 suppresses cell proliferation and cell cycle of ovarian cancer cells by selectively inhibiting CRM1. Our findings can potentially be translated towards clinical application of S109 against ovarian cancer.

## Materials and methods

### Cell culture, antibodies and reagents

The human ovarian carcinoma SKOV-3 and OVCAR-3 cells were maintained in RPMI-1640 medium supplemented with 10 % fetal bovine serum, 100 U/mL penicillin and 100 μg/mL streptomycin. S109 was synthetized by company. Antibodies against Actin, CRM1, RanBP1, IκB-α and flag tag were obtained from Santa Cruz Biotechnology (Santa Cruz, CA, USA). Antibodies against Foxo1, p27, p21, Cyclin D1, Cyclin B and Histone-H3 purchased from Cell Signaling Technology (CST, Beverly, MA). Alexa 488-conjugated donkey anti-rabbit antibody was obtained from Invitrogen Life Technology (Invitrogen, Carlsbad, CA).

### Cell viability assay

Cell proliferation was measured by a Cell Counting Kit-8 (CCK8) assay. Briefly, cells were seeded in quadruplicate on 96-well plates and incubated overnight under standard conditions to allow cell attachment. The cells were then treated with S109 in concentrations of 0 to 50 μM and incubated for 72 h. The MTT assay was performed by adding 10 μL of CCK8 to each well and incubating at 37 °C for 4 h. After incubation, the multiwell plates were then measured at 450 nm using a spectrophotometer.

### Immunofluorescence microscopy

SKOV-3 cells were seeded onto black optical-bottom 96-well glass plates and growth overnight. Medium was removed and replaced with drug-containing medium. Following the indicated treatments, cells were fixed for 20 min with 4 % formaldehyde in PBS at room temperature. Next, cell membranes were permeabilized by treatment with 0.3 % Triton X-100 in PBS for 20 min. After blocking with 1 % bovine serum albumin (BSA) in PBS for 1 h, cells were treated with primary antibodies (1:50 dilution) in blocking buffer. Fluorescent secondary antibodies anti-rabbit Alexa 488 were used at 1:200 dilution. After washing, cells were stained with 10 μg/mL DAPI. Photomicrographic images were acquired and analyzed using a fluorescence microscopy and photographed (Olympus, Japan).

### EdU assay

Cell proliferation was assessed by 5-ethynyl-2′-deoxyuridine (EdU) fluorescence staining using the Cell-Light™ EdU DNA Cell Proliferation Kit (Ruibo Biotech, Guangzhou, China) according to the manufacturer’s instructions [19]. The SKOV-3 cells were seeded in 96-well culture plates and incubated overnight. Then, the cells were treated with S109 at various concentrations (0, 1, 2, and 4 μM) for 12 h and incubated with 50 μM EdU for 4 h at 37 °C. Subsequently, the cells were fixed with 4 % paraformaldehyde for 15 min and then treated with 0.5 % Triton X-100 for 20 min. Thereafter, the cells were incubated with 100 μL of 1× Apollo® reaction cocktail for 30 min and then stained with DAPI for 15 min. After washing with phosphate-buffered saline (PBS) for 3 times, the cells were examined with fluorescence microscopy and photographed (Olympus, Japan).

### Cell clonogenic assay

SKOV-3 cells were seeded in six-well plates (600 cells/well) and treated with 0.1 % DMSO (vehicle) or S109 (1, 2 and 4 μM) for 12 h. After treatment, the drug-containing medium was removed and fresh medium was added to the wells. Medium was changed every 4 days for 10–14 days to allow for colony formation. Then, the cells were fixed with 4 % formaldehyde and stained with 0.1 % crystal violet solution. Finally, positive colony formations were manually counted.

### Cell cycle analysis

SKOV-3 cells were seeded in six-well plates at a density of 2 × 10^6^ cells per well and treated with 2 μM S109 for 24 h. After treatment, cells were collected and fixed in 70 % ethanol. Then, cells were washed twice with PBS and lastly stained with PI solution that contained 50 μg/mL PI and 25 μg/mL Rnase in the dark for 30 min. Subsequently, the cells was assayed on a FACSCalibur (Becton-Dickinson) and analyzed by CellQuest Pro software (Becton-Dickinson).

### Production of lentiviral and establishment of stable cell lines

The wild type or C528S mutation human CRM1 were cloned into a pWPXL lentiviral vector containing a sequence coding for a flag tag. The construct overexpress lentiviral vectors were co-transfected with pSPXA2 and pMD2.G plasmids into 293FT packaging cells using lipofectamine 2000 (Invitrogen). After 48 h incubation, the supernatant was collected and concentrated via ultracentrifugation. SKOV-3 cells were seeded into six-well plates and infected by CRM1-WT or CRM1-C528S lentivirals, respectively. After 48 h infection, the cells were continuously cultured in medium containing 2.5 μg/mL puromycin. The surviving cells were cultured into cell lines stably expressing CRM1-WT or CRM1-C528S.

### Western blotting

The whole or nuclear cell extract of control and treated cells were used in Western blot analysis [20]. The protein extracts were resolved by SDS-PAGE. After electrophoresis, proteins were electrotransferred to nitrocellulose membranes. The membrane was blocked and incubated with relevant antibodies. The proteins then were detected by enhanced chemiluminescence on X-ray film with an ECL Western blotting detection kit (Amersham).

### Data analysis

Data are means and standard deviations of three independent experiments with three to five replicates each. The results were statistical analyzed using a Student’s *t* test and considered statistically significant at the *p* < 0.05 level.

## Results

### Novel nuclear export inhibitor S109 suppresses CRM1 function in ovarian cancer cells

**Fig. 1:**
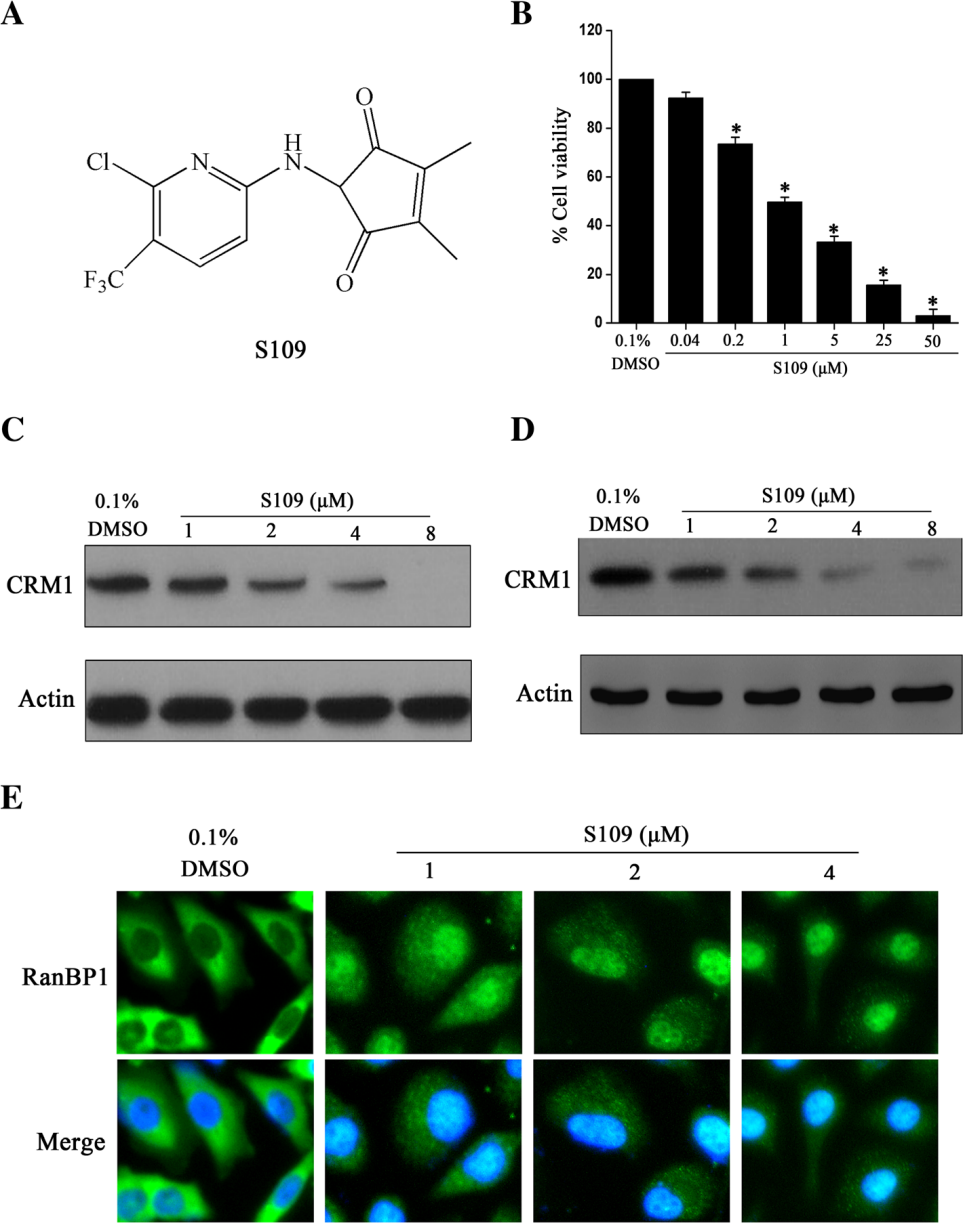
S109 inhibits ovarian cancer growth and RanBP1 nuclear export. **a** Structure of S109. **b** Suppression of the cell of growth of ovarian cancer cells by S109. The growth of SKOV-3 cells were cultured with indicated doses of S109 for 72 h and analyzed by the CCK-8 assay. All data are presented as the mean ± SEM of three replicates (*P < 0.05). **c** S109 reduces expression level of CRM1 protein. SKOV-3 cells were treated with indicated doses of S109 for 12 h, and the whole cell lysates were analyzed by immunoblotting. **d** S109 reduces expression level of CRM1 protein in OVCAR cells. Cells were treated with indicated doses of S109 for 12 h, and the whole cell lysates were analyzed by immunoblotting. **e** S109 inhibits nuclear export of RanBP1. Cells were treated with indicated doses of S109 for 2 h. Fixed cells were stained for RanBP1 and DAPI and analyzed by fluorescence microscopy

We have previously designed S109 as a new inhibitor of CRM1 (Fig. 1a). To investigate whether S109 is able to functionally inactivate CRM1 in ovarian cancer cells, we analyzed the subcellular localization of CRM1 cargo protein RanBP1, which is a canonical biomarker for CRM1 inhibition. As shown in Fig. 1e, RanBP1 is found exclusively in the cytosol in control cells. In contrast, treatment with S109 only 2 h led to a clear and rapid shift of RanBP1 to nucleus in a dose dependent manner. Next, the effect of S109 on the expression level of CMR1 protein in SKOV-3 and OVCAR-3 cells was analyzed. The level of CRM1 protein expression became markedly reduced in a dose-dependent manner on treatment with S109 (Fig. 1c and d). In order to investigate the effect of S109 on cell growth, we evaluated the cell viability of SKOV-3 cells treated with S109 for 72 h using the CCK-8 assay. As shown in Fig. 1b, S109 inhibits cell growth in a dose dependent manner. Notably, the cellular activities of S109 are consistent with their abilities to suppress nuclear export.

### The inhibitory effect of S109 is reversible

**Fig. 2:**
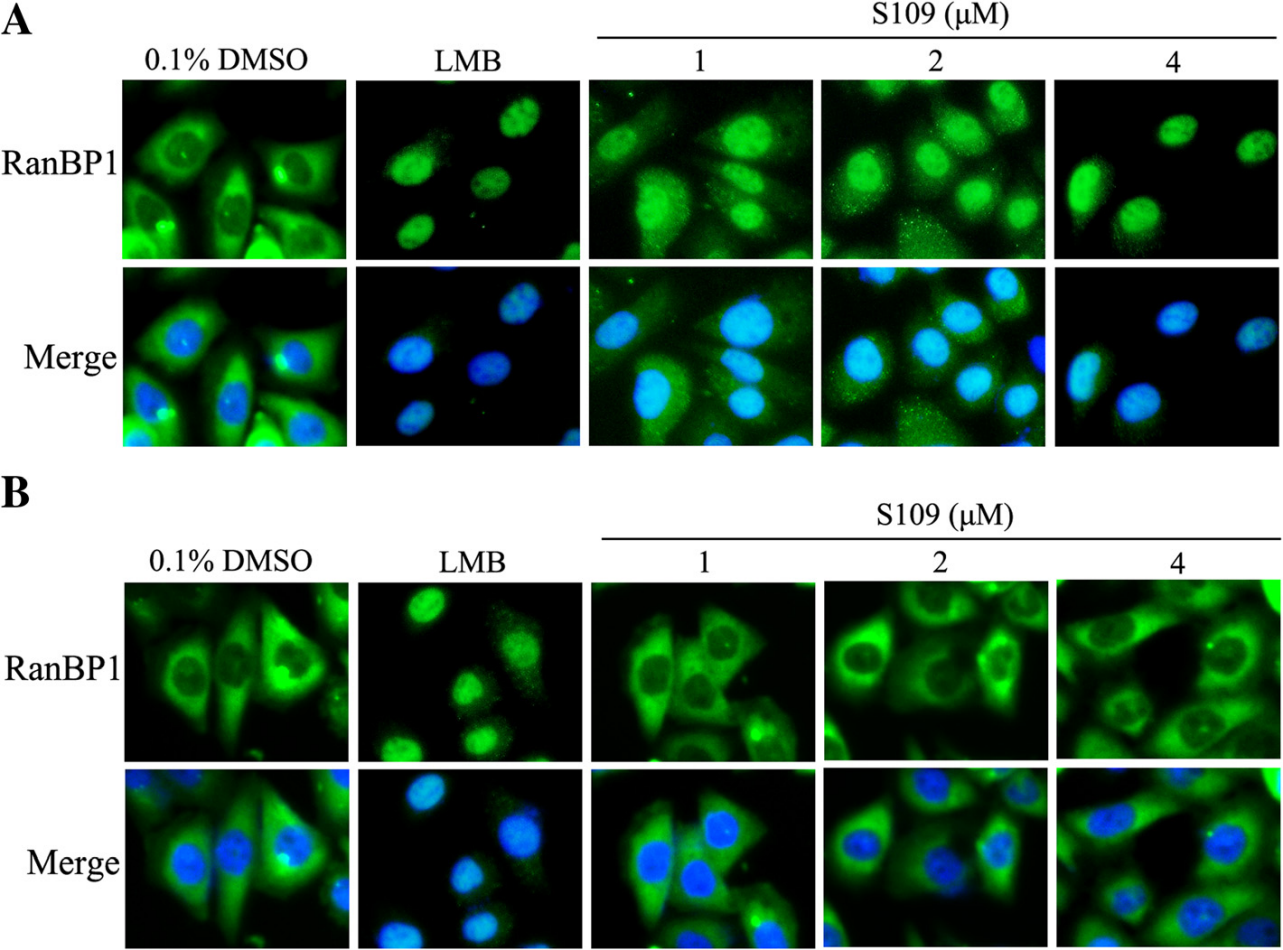
The inhibitory effects of nuclear export by S109 is reversible. **a** S109 and LMB inhibit nuclear export of RanBP1. SKOV-3 Cells were treated with indicated doses of S109 or LMB for 2 h. Fixed cells were stained for RanBP1 and DAPI and analyzed by fluorescence microscopy. **b** Reversible effect of S109 on the localization of RanBP1. Cells were incubated with indicated doses of S109 or LMB. After 2 h, the drugs were washed out and fresh medium was added. Cells were incubated for 1 h and then analyzed by fluorescence microscopy

We investigated whether the inhibition of CMR1 by S109 could be restored upon removal of the compound. Therefore, we incubated SKOV-3 cells with LMB or S109 for 2 h. In cells incubated with S109 or LMB, most of RanBP1 shifted from a cytoplasmic localization to nucleus, whereas in untreated cells, RanBP1 was still in the cytoplasm (Fig. 2a). Then, the cells were washed with PBS to remove the S109 or LMB, and new medium was added. After 1 h incubation, cells were monitored for RanBP1 localization by fluorescence microscopy. RanBP1 in cells incubated with S109 was almost completely translocated to the cytoplasm, whereas in cells incubated with LMB, RanBP1 still localized in the nucleus of the cells (Fig. 2b). Therefore, in contrast to LMB-treated cells, the majority of S109-treated cells were able to reverse completely the inhibitory effects of the drug.

### S109 inhibits the proliferation and colony formation of SKOV-3 cells

**Fig. 3:**
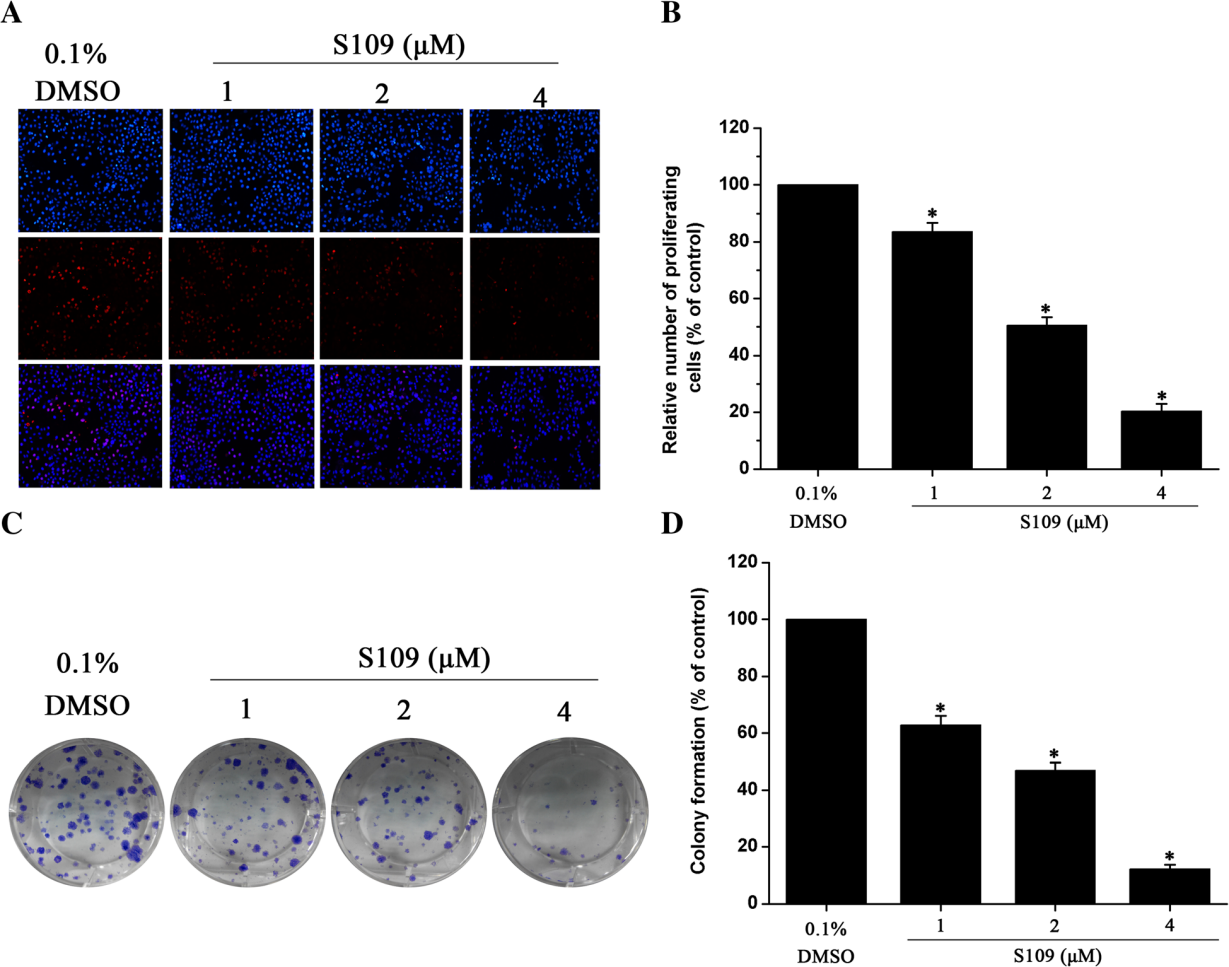
S109 inhibits proliferation and colony formation of SKOV-3 cells. **a** Representative EdU analysis of cell proliferation after S109 treatment. **b** Quantitativeresults of EdU incorporation assay. **c** S109 inhibits the colony formation of SKOV-3 cells. **d** Quantitative results of clonogenic assay. The percentage of proliferative cells or colony formation were normalized to that of the control group. All the data are presented as mean ± SEM in three repeats (*P < 0.05)

To further confirm the effects of S109 on cell proliferation, EdU fluorescence staining and clonogenic assays were performed. S109 treatment resulted in a significant reduction of the mean percentage of positive proliferative cells compared with control group (Fig. 3a and b). At 2 μM of S109, the proliferation was reduced to about 50.6 %. Since CCK-8 and EdU assay is a short term assay for proliferation and does not effectively convey cell survival, we performed a cell survival clonogenic assay to evaluate the long term effect of CRM1 inhibition. Figure 3c and d also show a dose dependent inhibition of clonogenic potential by S109 in SKOV-3 cells. The results from the clonogenic assay were consistent with the CKK-8 and EdU assay data shown suggesting that S109 inhibited cell growth in ovarian cells.

### S109 induces cell cycle arrest through nuclear localization of tumor suppressor proteins

**Fig. 4:**
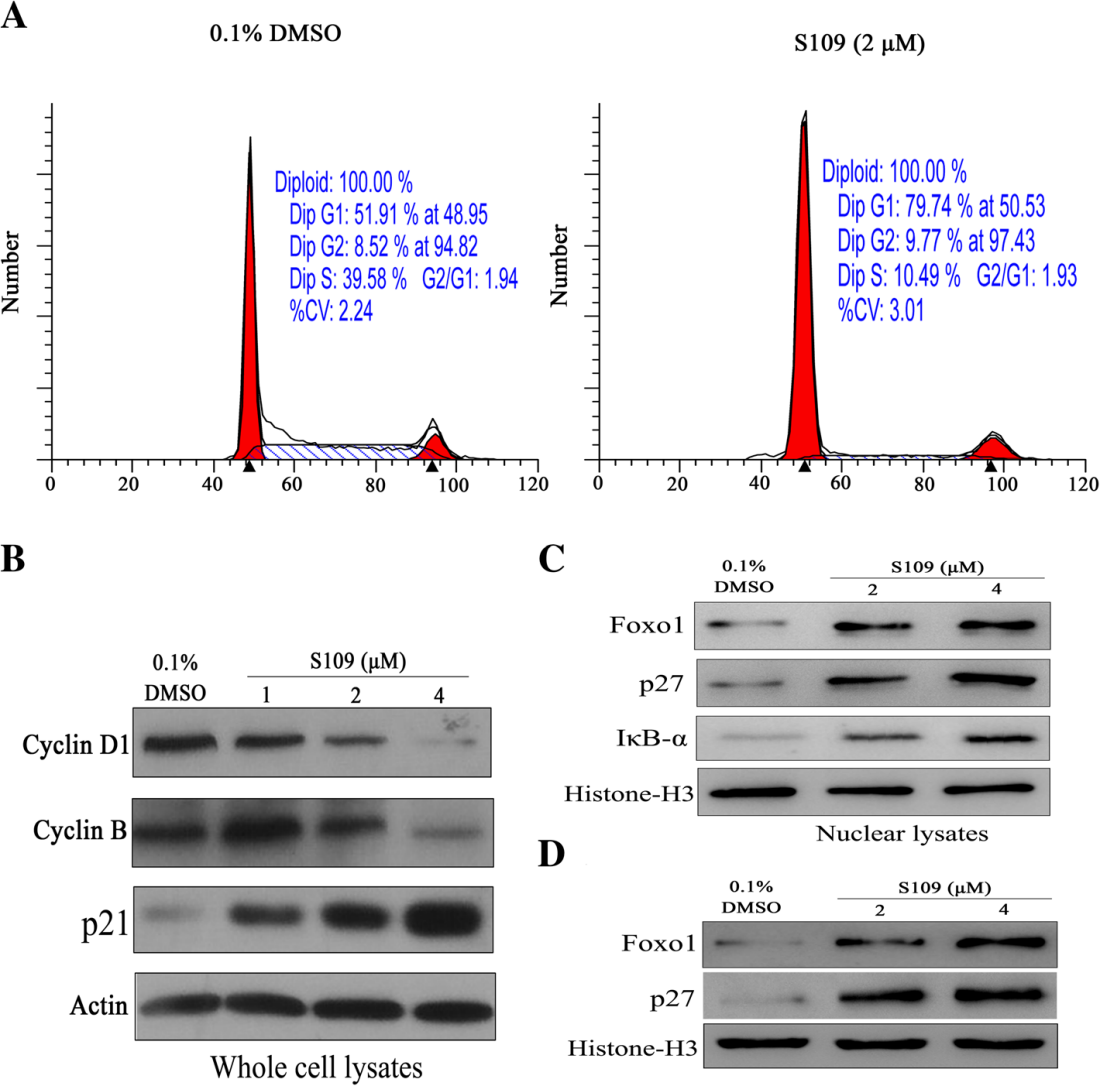
S109 induces cell cycle arrest and nuclear retention of tumor suppressor proteins. **a** SKOV-3 cells were exposed to 2 μM of S109 for 24 h. Cells were harvested, stained with propidium iodide and analyzed by flow cytometry. **b** SKOV-3 cells were treated with S109 at the indicated concentrations for 24 h. Cells were then harvested and subjected to immunoblot analysis. **c** SKOV-3 cells were treated with S109 at the indicated concentrations for 24 h. Nuclear proteins was extracted and subjected to immunoblot analysis. **d** OVCAR-3 cells were treated with S109 at the indicated concentrations for 24 h. Nuclear proteins was extracted and subjected to immunoblot analysis

In order to verify whether S109 works by abrogating cell cycle progression, propidium iodide flow cytometry assays were performed. SKOV-3 cells were exposed to 2 μM of S109 for 24 h and analyzed for cell cycle distribution. Our data clearly demonstrated cell cycle arrest at G1 phase in response to treatment with S109 as shown by an increase in the G1fraction from 51.9 % in the control cells to 79.7 % in S109-treated cells (Fig. 4a). A concomitant decrease in the percentage of S109-treated cells with respect to controls in S phase was observed. However, there were no significant effect of S109 treatment on the apoptosis of SKOV-3 cells (data not show). Together, these data demonstrate that the antiproliferation effects of S109 treatment are due to inducing cell cycle arrest, and not induction of apoptosis.

In order to delineate the molecular mechanisms of ovarian cancer cell cycle arrest induction by S109 in greater detail, the expression and nuclear localization of tumor suppressor proteins was investigated using western blot analysis. First, we measured the protein expression levels of cell cycle regulators after treatment with S109 by Western blotting with respect to the controls (Fig. 4b). Strikingly, we found a strong up-regulation of tumor-suppressor protein p27 in SKOV-3 cells upon treatment with S109. Furthermore, the expression of proliferative proteins Cyclin D1 and Cyclin B were downregulated in a dose-dependent manner after treatment with S109. Next, we investigated the effects of S109 on the nuclear accumulation of tumor suppressor proteins in ovarian cancer cells. As shown in Fig. 4c and d, exposure of SKOV-3 and OVCAR-3 cells to increasing concentrations of S109 resulted in a progressive increase in the nuclear fraction of major tumor suppressor proteins (Foxo1, p27 and IκB-α). Together, these data indicate that S109-induced cell cycle arrest is associated with downregulation of proliferative proteins and nuclear accumulation of tumor suppressor proteins.

### Mutation of CRM1 abolishes S109 cytotoxicity in ovarian cancer cells

**Fig. 5:**
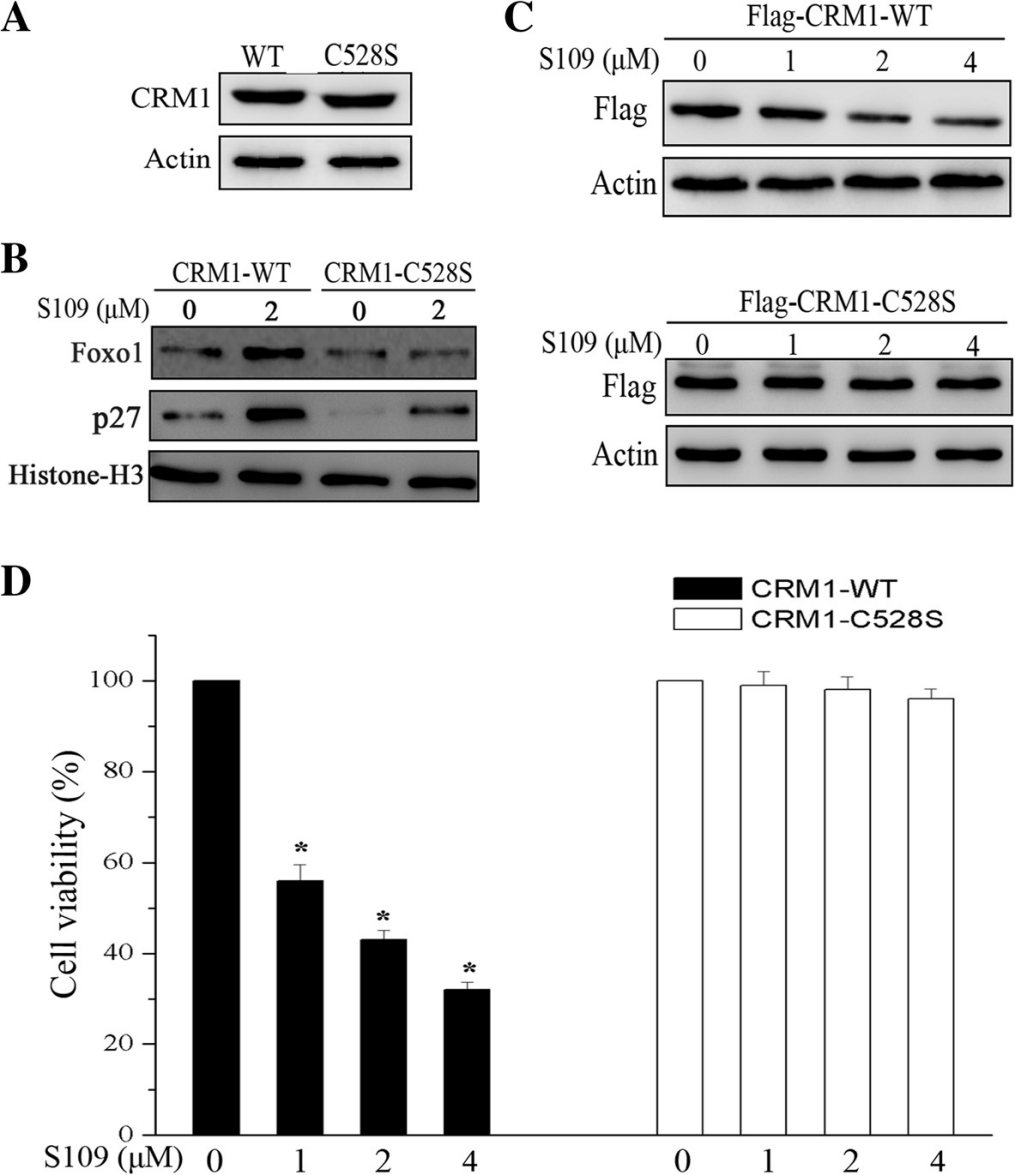
Cys528 mutation of CRM1 abolished the ability of S109 to inhibit proliferation. **a** Whole cell lysates were extracted from the cells stable expressing wild type or C528S mutant CRM1. Western blot analyses the expression level of CRM1 in both SKOV-3 cells. **b** Wild type and C528S mutant SKOV-3 cells were treated with S109 at the indicated concentrations for 24 h. Nuclear proteins was extracted and subjected to immunoblot analysis. **c** Wild type and C528S mutant SKOV-3 cells treated with indicated doses of S109 for 12 h. The whole cell lysates were analyzed by immunoblotting. **d** Growth inhibition assay in wild type and C528S mutant cells. Cells were seeded in 96-well plates and incubated with S109 at indiated concentrations for 72 h. Growth inhibition was analyzed by the CCK-8 assay. All the data are presented as mean ± SEM in three repeats (*P < 0.05)

LMB, the very potent inhibitor of CRM1, selectively binds to Cys528 of CRM1 [21]. To investigate whether the nuclear export inhibition of S109 is also dependent on the Cys528 of CRM1, we have prepared SKOV-3 cells stable expressing a wild type or C528S mutant CRM1. First, overexpression of wild or mutant type CRM1 did not alter the expression levels of CRM1 (Fig. 5a). However, in CRM1-C528S expressing SKOV-3 cells, exposure to S109 did not induce significant nuclear accumulation of tumor suppressor proteins, Foxo1 and p27 (Fig. 5b). Next, we analyzed the effect of S109 on the level of wild type or mutant CMR1 expression. As shown in Fig. 5c, cells expressing CRM1-C528S were resistant to S109, as CRM1 expression level did not show significant decrease even at high concentrations of S109.

We further evaluated whether S109 loses its ability to inhibit the proliferation of CRM1-C528S expressing cells. Consistent with our previous results, S109 treatment resulted in a significant growth inhibition at 1, 2 and 4 μM concentrations in CRM1-WT expressing cells. However, in CRM1-C528S expressing SKOV-3 cells, exposure to S109 did not induce significant growth inhibition at the same concentrations (Fig. 5d). Thus, based on these experimental observations, we have determined that the anticancer effects of S109 are dependent on binding to the Cys528 of CRM1.

## Discussion

Ovarian cancer is the most fatal among gynecololgic cancers [22]. Until now, however, there are no effective targeted treatment strategies for ovarian cancer. There is an urgent need for newer and more effective drugs for ovarian cancer treatment. In this report we show the anticancer potential of a novel small molecule CRM1 inhibitor S109 in ovarian cancer. S109 inhibits CRM1-dependent nuclear export, causing arrest of the cell cycle, inhibiting proliferation and suppressing clonogenic potential of ovarian cancer cells.

Recently, it has been reported that overexpression of CRM1 is correlated with poor prognosis in ovarian cancer [11]. Multiple tumor suppressor proteins are mislocalized or expressed at supraphysiogic levels within cancer cells [23]. CRM1 overexpression results in constitutive nuclear export of tumor suppressor proteins thereby facilitating proliferation [24]. Inhibition of nucleo-cytoplasmic transport by natural and synthetic compounds has been pursued as a therapeutic avenue in cancer based on a number of biologic observations [18]. Therefore, targeted inhibition of CRM1 is an attractive strategy against ovarian cancer.

Several CRM1 inhibitors have been described, however, most of them are irreversible inhibitors. We demonstrated that S109 could induce nuclear accumulation of RanBP1, a canonical biomarker for CRM1 inhibition. This effect was clearly and quickly reversible in the majority of the cells, whereas the inhibitory effect of LMB could not be reversed. These results indicate that S109 is a reversible CRM1 inhibitor in ovarian cancer cells. We also found that S109 reduced CRM1 protein levels in ovarian cancer cells. However, the irreversible inhibitor LMB could not induce decrease of CRM1 [25]. Most of CRM1 inhibitors have antitumor effects without affecting the protein level of CRM1. The antitumor effects of S109 may be not due to the inhibition of CRM1 down-regulation by S109. We argued that although reversible inhibitors bind to the same residue in CRM1 as LMB, reversible inhibitors can likely change the conformation of CRM1 such that it is recognized by the proteasomal degradation machinery and thereby degraded.

The PI3K/Akt pathway plays an important part in the regulation of multiple biological processes, including apoptosis, metabolism, cell proliferation and cell growth [26]. It is one of the pathways most frequently targeted by genomic aberrations in ovarian cancer. There is growing evidence that genetic degergulation of key components of the PI3K/Akt pathway results in the activation of downstream signaling pathways, including Foxo1, Cyclin D1, Bad and NF-κB [27]. These Akt pathway components might be therapeutic targets and important mediators of resistance to other targeted therapies. Thus, a therapeutic strategy simultaneously targeting multiple oncogenic pathways or targets might be a rational and effective strategy in ovarian cancer. CRM1 inhibitors can target multiple oncogenic pathways simultaneously [18]. We found that S109 could downregulate the expression of Cyclin D1 and Cyclin B, and induce nuclear retention of major tumor suppressor proteins (Foxo1, p27 and IκB-α). These tumor suppressor proteins play important roles in Akt, NF-κB and cell cycle pathway, respectively.

Cytoplasmic Foxo1 has been shown to be particularly high expression both in paclitaxel-resistant ovarian cancer cell lines and clinical samples [28]. In contrast, nuclear Foxo1 is a favorable factor and induced by paclitaxel treatment. Foxo1 could be a molecular target for the treatment of sensitive or drug-resistant ovarian cancers. Our data demonstrated that S109 could lead to retention of Foxo1 in the nucleus, contributing to cell cycle arrest. Our results suggest that foxo1 is an important component of the downstream signaling pathway of CRM1 inhibition in ovarian cancer cells.

The present study shows for the first time that S109 has prominent antitumor activity against ovarian cancer cells. S109 reduced levels of CRM1 protein and inhibited cell proliferation. In particular, we have provide strong evidence in support of a S109 mechanism of action that involves nuclear retention of different tumor suppressor proteins, especially Foxo1. Together, our study identifies CRM1 as a novel target in ovarian cancer and demonstrates that S109 can act as potent agents for ovarian cancer treatment.
